# Exploring the impact mechanism of low-carbon multivariate coupling system in Chinese typical cities based on machine learning

**DOI:** 10.1038/s41598-023-31590-z

**Published:** 2023-03-20

**Authors:** Haonan Yang, Liang Chen, Huan Huang, Panyu Tang, Hua Xie, Chu Wang

**Affiliations:** 1 School of Finance and Accounting, Chengdu Jincheng College, Chengdu, 610097 China; 2 Postdoctoral Station of Management Science and Engineering, Chengdu University of Technology, Chengdu, 610059 China; 3 College of Business, Chengdu University of Technology, Chengdu, 610059 China

**Keywords:** Environmental economics, Urban ecology

## Abstract

Low-carbon city construction is one of the key issues that must be addressed for China to achieve high-quality economic development and meet the Sustainable Development Goals. This study creates a comprehensive evaluation index system of low-carbon city multivariate system based on carbon emission data from 30 typical Chinese cities from 2006 to 2017 and evaluates and analyzes the trend of city low-carbon levels using the CRITIC-TOPSIS technique and MK method. Meanwhile, the influence mechanism of the multi-coupled system is investigated using the coupling coordination degree model and random forest algorithm.The results show that there are 8 cities with a significant increasing trend of low-carbon level, 19 cities with no significant monotonic change trend, and 3 cities with a decreasing trend of low-carbon level. By analyzing the coupling coordination degree, we found that the coupling coordination degree between low-carbon level and economic development in most cities tends to increase year by year, from the initial antagonistic effect to a good coordination development trend, which confirms the “inverted U-shaped” relationship between economy and carbon emission. In addition, industrial pollutant emissions, foreign direct investment, and economic output are the core drivers of low-carbon levels in cities.

## Introduction

Currently, the climate problem has had varying degrees of negative impacts on countries around the world^1^, and natural disasters caused directly or indirectly through various means have hampered local economic and social development, as well as had significant impacts on residents’ livelihoods and health. Among these natural disasters caused by climate change, global warming caused by a significant increase in greenhouse gases and its subsequent natural disasters has received the most attention^2^. Since the Kyoto Protocol in 1997, greenhouse gas emissions have become a hot topic of global concern, and many scholars have conducted studies on the subject^3,4^, the most influential of which is the well-known Stern Review^5^. One of the review’s central ideas is that humans should immediately and significantly reduce greenhouse gas emissions to address the threats to human survival posed by climate change. Although some of the review’s assumptions have been criticized in academic circles^6,7^, the review’s positive advocacy and promotion of global greenhouse gas emission reduction cannot be overlooked. Carbon dioxide emission reduction has been a major concern as the most common greenhouse gas, and various studies related to carbon dioxide emissions have increased, for example, to reduce the level of green premium brought by low-carbon technologies, many researchers and scholars have put forward their views on carbon taxes and carbon emissions trading^8,9^. Furthermore, to analyze the global greenhouse gas reduction problem, some studies have used carbon dioxide as a uniform standard for accounting for greenhouse gases^10^.

For China, the international community and academia have been keeping a close eye on Chinese CO$_2$ emissions and when they will peak^11,12^. China stated in the Sino-US Joint Statement on Climate Change released in 2014 that its CO$_2$ emissions would peak by 2030^13^. The following year, China announced its post2020 emissions reduction targets, intending to reach a peak in CO$_2$ emissions by 2030 or earlier^14^. Based on the foregoing, it has become an urgent task for the Chinese government to implement effective climate policies to reduce domestic CO$_2$ emissions. According to statistics, cities account for more than 70 % of global CO$_2$ emissions^15^. Higher levels of urbanization have a two-fold effect on total urban carbon emissions, which is also consistent with the Environmental Kuznets Curve hypothesis (EKC)^16^. On the one hand, increased urbanization will result in more non-construction land being converted into construction land in cities, resulting in a corresponding change in urban spatial structure. On the other hand, increased urbanization will promote scientific and technological progress, resulting in the transformation and upgrading of industries, as well as the corresponding adjustment and upgrading of urban industrial structures, and the emergence of industrial structures that match the spatial functions of cities and towns, thereby reducing energy consumption and further reducing carbon emissions^17,18^.This demonstrates that the achievement of carbon peaks by Chinese cities is central to achieving a national carbon peak. In the study of carbon emissions from various cities, megacities with higher economic and industrialization levels play an important role in the overall goal’s achievement.

In the process of exploring urban CO$_2$ emission reduction pathways, how to balance economic development and ecological sustainability is an issue that must be considered in pathway design and implementation, in addition to solving the problem of high urban carbon emissions. The relationship between economic development and the ecological environment is commonly thought to follow the EKC hypothesis^19^, which states that economic development only pollutes and degrades the environment at the beginning and that after a certain per capita income level is reached, economic development has a positive effect on the environment^20,21^.Meanwhile, the EKC hypothesis also suggests a similar nonlinear inverted U-shaped relationship between economic development and urban carbon dioxide emissions, i.e., urban carbon emissions are positively correlated with economic development at the initial stage of economic growth and then decline when economic development reaches a certain level^22^. In addition, some scholars have obtained results through empirical analysis that do not support the EKC hypothesis^23^, while others argue that there is an N-shaped relationship between economic growth and carbon emissions^24^.

**Figure 1:**
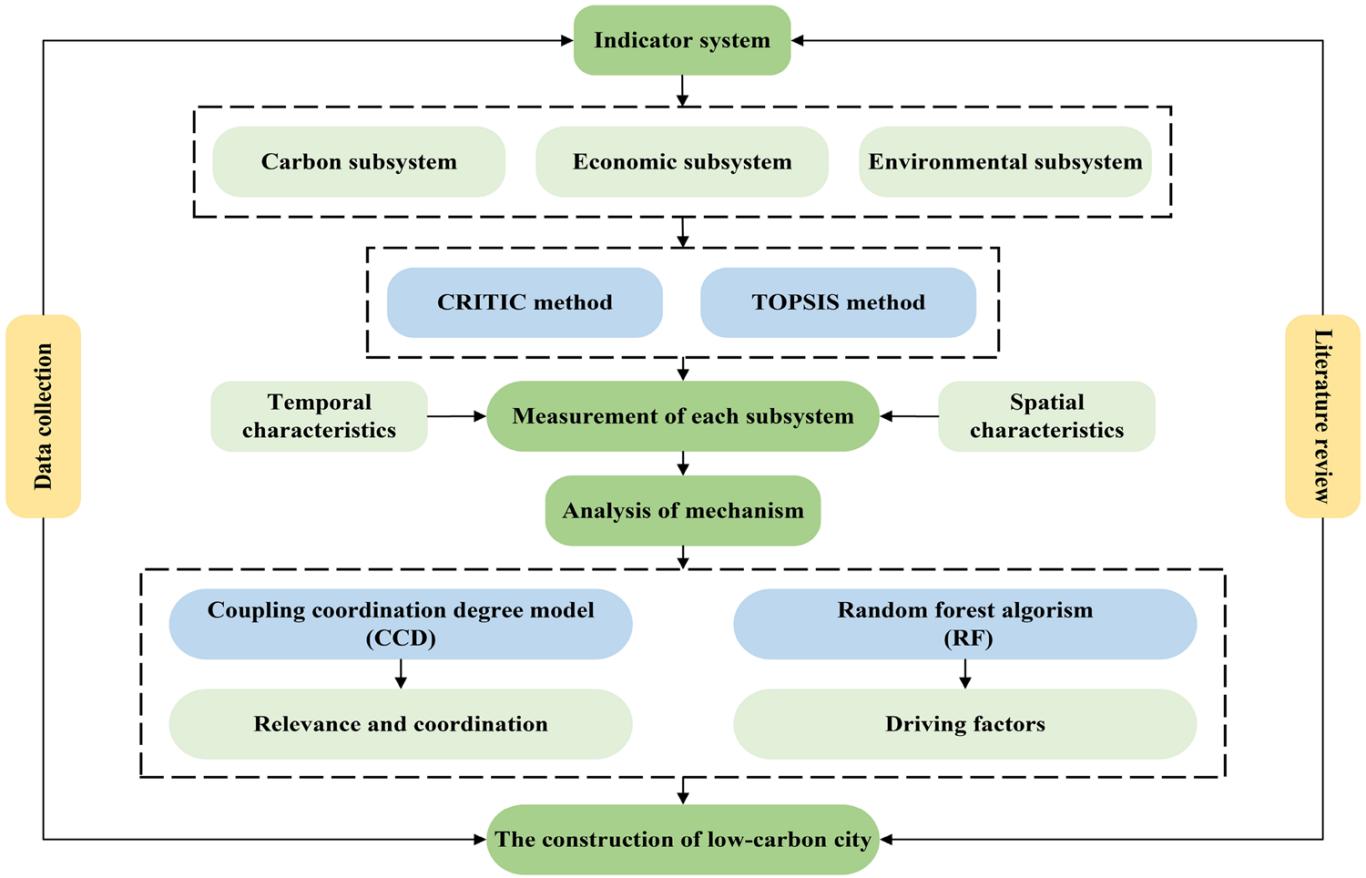
Research path.

As mentioned above, China is one of the world’s largest emitter of carbon dioxide emissions, and urban carbon emissions are the most important source of national carbon emissions. Therefore, this study selects typical cities in China as the research object, and investigates the interactions and influence mechanisms of urban multi-systems, aiming to provide new references and inspirations for the construction of low-carbon cities in China and even globally. Most of the existing studies on low-carbon cities focus on the EKC hypothesis, and regression analysis is conducted on single or multiple variables related to CO$_2$ emissions by constructing various econometric models. Most of the studies focus on macro mechanisms at the national or provincial level, but there are few studies on low-carbon level measurement and impact mechanisms based on prefecture-level cities. At the same time, the solutions to specific problems are relatively old, and it is difficult to conduct a complete and systematic search for the core drivers that influence the trend of low-carbon levels in cities. Because of this, to analyze the coupling coordination and mechanism between urban carbon emission, economy, and environment, and to explore the key driving factors affecting urban carbon emission, this research constructs a multifaceted indicator system consisting of a low-carbon subsystem, socio-economic subsystem, and ecological environmental subsystem, and combines statistics, machine learning, and other frontier sciences to study the coupling and coordinated development of low-carbon cities in typical Chinese cities. The research also explores and analyzes the core driving factors affecting the low-carbon level of cities, and analyzes the causes of the mechanism of action in the context of the actual situation of Chinese development. In addition, based on social reality, this research deconstructs the multi-system mechanism of low-carbon cities from the perspectives of the economy and government system, and expands the ideas of low-carbon city construction and development, to expect a breakthrough in measuring the status of low-carbon construction in cities, analyzing the trend of low-carbon level changes in cities, analyzing the multi-system coupling and coordination mechanism, and exploring the core drivers of sustainable urban development, etc. The specific research path is shown in the following figure. (Fig. 1 ).

## Literature review

Since 2009, the term “low-carbon city” has gradually gained traction in a variety of fields such as academic research, policy formulation, and urban planning^25^. At the moment, academic research for low-carbon city construction can be roughly divided into three categories: policy assessment, comprehensive evaluation, and mechanism analysis. Most established studies on policy evaluation in Chinese cities have used the Differences-in-Differences method (DID) of econometric models, primarily to empirically examine changes in the variables of interest to the research before and after the implementation of the Low-Carbon City Pilot (LCCP)^26^ and the Carbon Emission Trading Scheme (CTS) policies in China.Carbon emission reduction^27,28^, carbon emission efficiency^29^, energy efficiency^30^, and urban green total factor productivity (GTFP)^31,32^ are currently studied variables. According to the studies mentioned above, the DID approach has been widely used in China to evaluate policies related to low-carbon city construction for a variety of scenarios. This is because the DID series of methods’ principles are relatively simple to grasp and can alleviate the endogeneity problem to some extent, so the method has gained popularity among academics in recent years .

At present, many cities around the world are promoting low-carbon development, but due to the difference in resource-carrying capacity in different regions, there are certain differences in the development status between different regions^33^. Establishing a scientific and effective comprehensive indicator system, can help decision-makers to better quantify the low-carbon levels in different regions, and facilitate the overall control of urban low-carbon levels. In the study of a comprehensive evaluation of low-carbon cities, it is first necessary to consider how to construct a scientific and reasonable low-carbon city index system. Social, economic, cultural, and other factors must be fully considered to establish an index evaluation system that matches the local development conditions^34^. The current research mainly starts from the dimensions of urban economy, society, energy, carbon emissions, ecological environment, and urban transportation when constructing a low-carbon city comprehensive evaluation system^35,36^, and completes the framework construction of the index system. To sum up, the framework construction of the index system by the predecessors has been relatively mature. Therefore, in the process of constructing the evaluation index system, this research refers to the research of the predecessors and starts from the three dimensions of urban carbon emissions, economic development, and environment to construct a typical Chinese model. Urban multi-system evaluation index system.

At the same time, the method of a comprehensive evaluation is commonly used for relevant research in the current process of measuring and analyzing urban low-carbon levels. The current mainstream index weight distribution methods can be roughly divided into three categories in terms of weighting method selection: subjective weighting, objective weighting, and subjective-objective combination weighting. Although combined weighting can reduce information loss and bring the weight value closer to reality, it has stringent requirements for the rationality and dependability of subjective weight distribution results. In many studies, the objective weighting method is still used for weight distribution.For example, for objective weight distribution, use the entropy method^37^, or for weight distribution, use the CRITIC method^38^. The most common comprehensive evaluation models are the fuzzy comprehensive evaluation method^39^ for the fuzzy index environment, and the TOPSIS method^40^ and VIKOR method^41^ for solving multi-criteria decision-making (MCDM) problems^42^.The advantage of the TOPSIS method is that it can make full use of the properties of the evaluation objects and determine the distance between each evaluation object and the positive and negative ideal solutions as well as their corresponding relative posting schedule^43^. As a result, this method is widely used in evaluating various index systems. In a thorough examination. Existing studies typically use the entropy-TOPSIS method for comprehensive evaluation and analysis to avoid subjective factors weakening the objectivity of index weights^44^. Given this, this research combines the objective weighting method CRITIC with the comprehensive evaluation method TOPSIS to conduct a comprehensive evaluation and comparative analysis of 30 typical Chinese urban multi-systems, providing a reference for the subsequent comprehensive evaluation of low-carbon cities..

Existing studies on the impact mechanism of low-carbon cities have mostly borrowed the environmental Kuznets curve to investigate the economy-environment, economy-low-carbon, and environment-low-carbon subsystems. To investigate the mechanism between the subsystems, determine whether the inverted U-shaped hypothesis is satisfied. Simultaneously, some scholars established an evaluation index system for the city and used a comprehensive evaluation model to evaluate each subsystem thoroughly. The coordination degree model investigates the mechanisms that exist between various subsystems^45,46^. Furthermore, a significant number of researchers use spatial econometric models to investigate the factors influencing urban low-carbon levels and the degree of spatial aggregation^47,48^.

In relevant academic research on the core driving factors of urban carbon emissions, the Kaya identity was first used to describe the relationship between carbon emissions and energy efficiency, energy structure, economic level, and population size^49^.In the subsequent analysis of the driving factors of carbon emissions, an index decomposition method based on the Laspeyres method and the Divisia method was developed^50,51^ to decompose the variable of carbon dioxide emissions into population, economy, energy, technology, etc. A range of variables was analyzed to explore the core drivers of CO$_2$ emissions^52^.With the popularity of cutting-edge subject methods such as machine learning in recent years, some scholars have proposed using random forest and other methods to analyze the core driving factors of carbon emissions, providing new methods and research ideas for determining the core driving factors of carbon emissions^53^.Therefore, in the selection of the exploration and analysis method for the core driving factors, this research draws on the random forest algorithm adopted by the existing research, takes the total carbon dioxide emission of the city as the dependent variable, and takes the indicators of the economic subsystem and the environmental subsystem as the dependent variable. Independent variables, using the random forest algorithm (RF), construct multiple regression decision tree models and explore and study the core driving factors of carbon emissions in typical Chinese cities.

To summarize, the coupling coordination degree model is mostly used for the mechanism analysis of urban multivariate systems, but there have been few studies on its combination with machine learning algorithms. As a result, based on the comprehensive evaluation results of typical Chinese cities, this research combines the coupling coordination model with the machine learning algorithm model to further investigate the coupling coordination and core driving factors between urban multi-systems and provides a reference for subsequent analysis of urban multivariate systems.

## Materials and methods

### Study area

**Figure 2:**
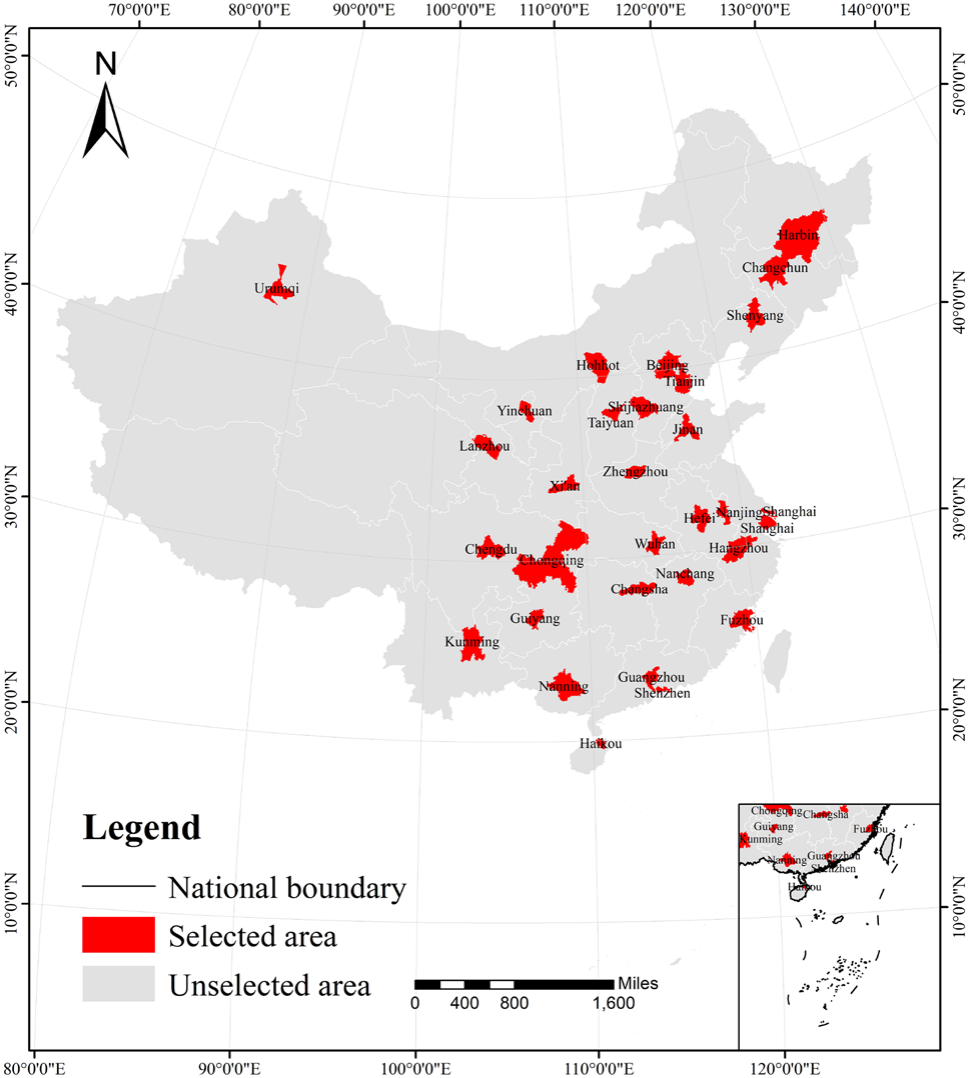
Study area.

This study focuses on the measurement of carbon emission levels and the influence mechanism of quintessential Chinese cities. 30 representative large cities in seven regions of China are selected as the study area, specifically Shenyang, Harbin, and Changchun in Northeast China, Beijing, Tianjin, Shijiazhuang, Taiyuan, and Hohhot in North China, Shanghai, Nanjing, Hangzhou, Hefei and Fuzhou in East China, Guangzhou, Nanning, Shenzhen, and Haikou in South China, Zhengzhou, Wuhan, and Changsha in Central China, Chengdu, Chongqing and Fuzhou in Southwest China. In the south of China, Guangzhou, Nanning, Shenzhen, Haikou, Zhengzhou, Wuhan, and Changsha in central China, Chengdu, Chongqing, Kunming, and Guiyang in southwest China, and Xi’an, Lanzhou, Yinchuan, and Urumqi in northwest China (Fig. 2). The evaluation index system is constructed by analyzing and screening the indicators related to the carbon emission status, ecological and environmental quality, and economic development of cities, and realizing the comprehensive measurement of low-carbon level of 30 quintessential cities in the past 15 years as well as the analysis of the subsequent influence mechanism.

### Data sources

The raw data of CO$_2$ emissions from Chinese prefecture-level cities in this study were obtained from ground-based observations of CO$_2$ emissions at the county level in China, which using satellite remote sensing data were estimated by Chen et al.^54^, and published on Figshare^55^. By aggregating county-level data, this research obtains data at the prefecture-level city level. The original data for other environmental and economic subsystem-related indicators come from the “China City Statistical Yearbook” and “China Urban and Rural Construction Statistical Yearbook.” Per capita carbon dioxide emissions, and per GDP carbon dioxide emissions are calculated.

### Construction of evaluation indicator system

Using previous research foundations, this study will begin with the three dimensions of urban carbon emissions, economy, and environment, and will then construct a multi-system comprehensive evaluation index system for typical Chinese cities, selecting 30 typical Chinese cities. A comprehensive evaluation and analysis of the city’s urban multi-system is carried out using relevant data from 2006 to 2017.

Among them, in terms of urban carbon emissions, in addition to the total carbon dioxide emissions indicators, this research also selects the total carbon dioxide emissions per capita and carbon dioxide emissions per unit of GDP as the evaluation indicators of urban carbon emissions, so that population size and economic conditions are also included in the evaluation of carbon emissions The range of indicators considered. In the comprehensive evaluation of low-carbon cities, in addition to considering the emission of greenhouse gases, urban economic development, and people’s living conditions also need to be included in the evaluation index system^56^. In the selection of urban economic development indicators, GDP is the most commonly used indicator to measure the level of urban economic development^57^. Foreign direct investment (FDI) is also a hot issue in analyzing the impact of economic development on total carbon dioxide emissions. For developed countries, under normal circumstances, FDI can promote technology spillover effects, improve local production capacity, and relieve local employment pressure to a certain extent^58^, but for developing countries, since there is no right to choose a foreign investment, Therefore, the pollution produced by developed countries are forced to be transferred to some developing countries^59^. Furthermore, fiscal taxation of local governments is a relatively effective tool for local governments to regulate the macroeconomic environment, and it plays an important role in local finance, so it should be considered in the urban economy. The index system is generally constructed in three dimensions for the index selection of the urban environmental subsystem: solid, liquid, and gas. In this research, the annual average concentration of PM2.5, industrial smoke and dust emissions, industrial wastewater emissions, and industrial sulfur dioxide emissions are chosen as environmental subsystem evaluation indicators based on existing research^60^ (Table 1).

**Table 1:** Low-carbon city multi-system comprehensive evaluation indicator system.

Subsystem	Indicator	Unit	Type	Weight
Carbon subsystem (A)	Total CO$_2$ emission(A1)	Million ton	$-$	0.446
	Per capita CO$_2$ emission (A2)	Tons/person	$-$	0.232
	Per GDP CO$_2$ emission (A3)	Tons/million yuan	$-$	0.321
Economic subsystem (B)	GDP (B1)	Million yuan	+	0.342
	Foreign direct investment (B2)	Million yuan	+	0.374
	Local fiscal general budget revenue (B3)	Million yuan	+	0.284
Environmental subsystem (C)	PM2.5 annual concentrations (C1)	$\upmu {\text{g}}/{\text{m}}^{3}$	$-$	0.257
	Total industrial smoke(dust) emission (C2)	Million tons	$-$	0.348
	Total industrial wastewater discharge (C3)	Million tons	$-$	0.182
	Total industrial SO$_2$ emission (C4)	Million tons	$-$	0.213

### Method

#### CRTIC method

The CRITIC method is an objective weighting method, compared to the general entropy weighting method, which introduces a comprehensive consideration of the variability between elements and the conflict between elements while considering the information size of the indicators, and uses the form of product to reflect the information size within the data, and determines the objective weights of the indicators on this basis, as follows.

First, the standard deviation as well as the correlation coefficients of each element are calculated, thus reflecting the variability and correlation of each element of the comprehensive measure of low-carbon level in cities.

1 $$\begin{aligned} {S_j} = \sqrt{\frac{{\sum \nolimits _{i = 1}^n {{{({x_{ij}} - {{{\bar{x}}}_j})}^2}} }}{{n - 1}}} \end{aligned}$$

2 $$\begin{aligned} {R_j} = \sum \nolimits _{i = 1}^n {(1 - {r_{ij}})} \end{aligned}$$

Then, the weights of the elements are determined based on the calculation results (where *n* represents the number of research objects and *m* represents the number of indicators).

3 $$\begin{aligned} {w_j} = \frac{{{S_j}{R_j}}}{{\sum \nolimits _{j = 1}^m {{S_j}{R_j}} }} \end{aligned}$$

#### TOPSIS comprehensive evaluation model

The sorting principle of the TOPSIS comprehensive evaluation method is to determine the positive and negative ideal solutions, and then calculate the distance between each evaluation object and the positive and negative ideal solutions and the corresponding relative progress, which are used as the basis for evaluating the degree of pros and cons. The specific formula is as follows.

4 $$\begin{aligned} {x_{ij}} = \frac{{{t_{ij}} - \min {t_{ij}}}}{{\max {t_{ij}} - \min {t_{ij}}}}\left( {If\;{t_{ij}}\;is\;a\;positive\;indicator} \right) \end{aligned}$$

5 $$\begin{aligned} {x_{ij}} = \frac{{\max {t_{ij}} - {t_{ij}}}}{{\max {t_{ij}} - \min {t_{ij}}}}\left( {If\;{t_{ij}}\;is\;a\;negative\;indicator} \right) \end{aligned}$$

$t_{ij}$ represents the *j*th indicator in the *i*th year, $t_{ij}$ represents the data value corresponding to the *j*th index in the *i*th year of the original data, $x_{ij}$ represents the data after standardization process.

After completing the standardization of the indicators by Eqs. (4) and (5), determine its positive and negative ideal solutions ${Sd}_j^+$ and ${Sd}_j^-$, and the relative closeness $\eta _j$. Among them, $p_j^+$ represents the maximum value of the *j*th index, $p_j^-$ represents the minimum value of the *j*th index, and $w_j$ represents the weight value of the *j*th index after the combined weighting.

6 $$\begin{aligned} Sd_i^ += & {} \sqrt{\sum \nolimits _{j = 1}^m {{{\left( {p_j^ + - {x_{ij}}} \right) }^2}} } \end{aligned}$$

7 $$\begin{aligned} Sd_i^ -= & {} \sqrt{\sum \nolimits _{j = 1}^m {{{\left( {p_j^ - - {x_{ij}}} \right) }^2}} } \end{aligned}$$

8 $$\begin{aligned} {\eta _j}= & {} \frac{{Sd_j^ - }}{{Sd_j^ + + Sd_j^ - }} \end{aligned}$$

#### Coupling coordination degree model

The coupling coordination degree model is a quantitative calculation model used to analyze the level of development of coupling coordination between systems of things^61^. The coupling coordination degree model involves the calculation of a total of several index values, which are the coupling degree value coupling coordination degree value. Where the coupling degree refers to the dynamic correlation between two or more systems that interact and influence each other to achieve coordinated development and can reflect the degree of interdependence and mutual constraints between systems^62^. Where the coupling degree is the indicator that evaluates the synergy of the indicators of the study, and if the coupling degree is larger, the stronger the synergy, which is expressed in the smaller size of the difference in the evaluation coefficients between the systems^63^. The coupling coordination degree refers to the size of the degree of benign coupling in the coupled interaction relationship, which can reflect the good or bad coordination status and is used to find out the positive development of the relationship between the systems^64^. The coupling coordination degree value and the coordination level classification criteria can be combined to finally arrive at the coupling coordination level degree of each item. The coupling coordination degree model is calculated as follows.

9 $$\begin{aligned} C= & {} n \times {\left[ {\frac{{{U_1}{U_2} \cdots {U_n}}}{{{{({U_1} + {U_2} + \cdots {U_n})}^n}}}} \right] ^{\frac{1}{n}}} \end{aligned}$$

10 $$\begin{aligned} T= & {} \sum \nolimits _{i = 1}^n {{\alpha _i}{U_i}} \end{aligned}$$

11 $$\begin{aligned} D= & {} \sqrt{C \cdot T} \end{aligned}$$

where *C* denotes the coupling degree, $U_i$ denotes the index of the *i*th subsystem, *T* denotes the coordination degree, $\alpha _i$ denotes the weight coefficient of the *i*th subsystem, and this research assumes that $\alpha _i=\frac{1}{n}$, while *D* denotes the coupling coordination degree.

#### Random forest algorithm

The random forest algorithm is a bagging algorithm in the ensemble learning that is commonly used to solve classification and regression problems^65^.The Bootstrap method is used to generate multiple training subsets, and then a decision tree is created for each training subset for training, and finally, the results of each decision tree are combined to obtain the overall result. In terms of decision tree type, there are two options: classification decision tree and regression decision tree.Because the urban low-carbon index is characterized as a continuous variable rather than a discrete variable representing a category, this research chooses to build a regression-type decision tree and selects features and branches based on the MSE value (Eq. 12), which in order to complete the ranking of the importance of indicators. Among them, *a* denotes an arbitrary division feature, *s* denotes an arbitrary division point, and the original dataset is divided into two datasets, *D*_*1*_ and *D*_*2*_, by dividing the point *s*. *c*_*1*_ and *c*_*2*_ denote the mean values of the datasets *D*_*1*_ and *D*_*2*_, respectively, and *y*_*i*_ represents the feature values.

12 $$\begin{aligned} {\min _{a,s}}\left[ {{{\min }_{{c_1}}}\sum \limits _{{x_i} \in {D_1}} {{{\left( {{y_i} - {c_1}} \right) }^2} + {{\min }_{{c_2}}}\sum \limits _{{x_i} \in {D_2}} {{{\left( {{y_i} - {c_2}} \right) }^2}} } } \right] \end{aligned}$$

## Results

### Analysis of low-carbon subsystem measurement results

**Figure 3:**
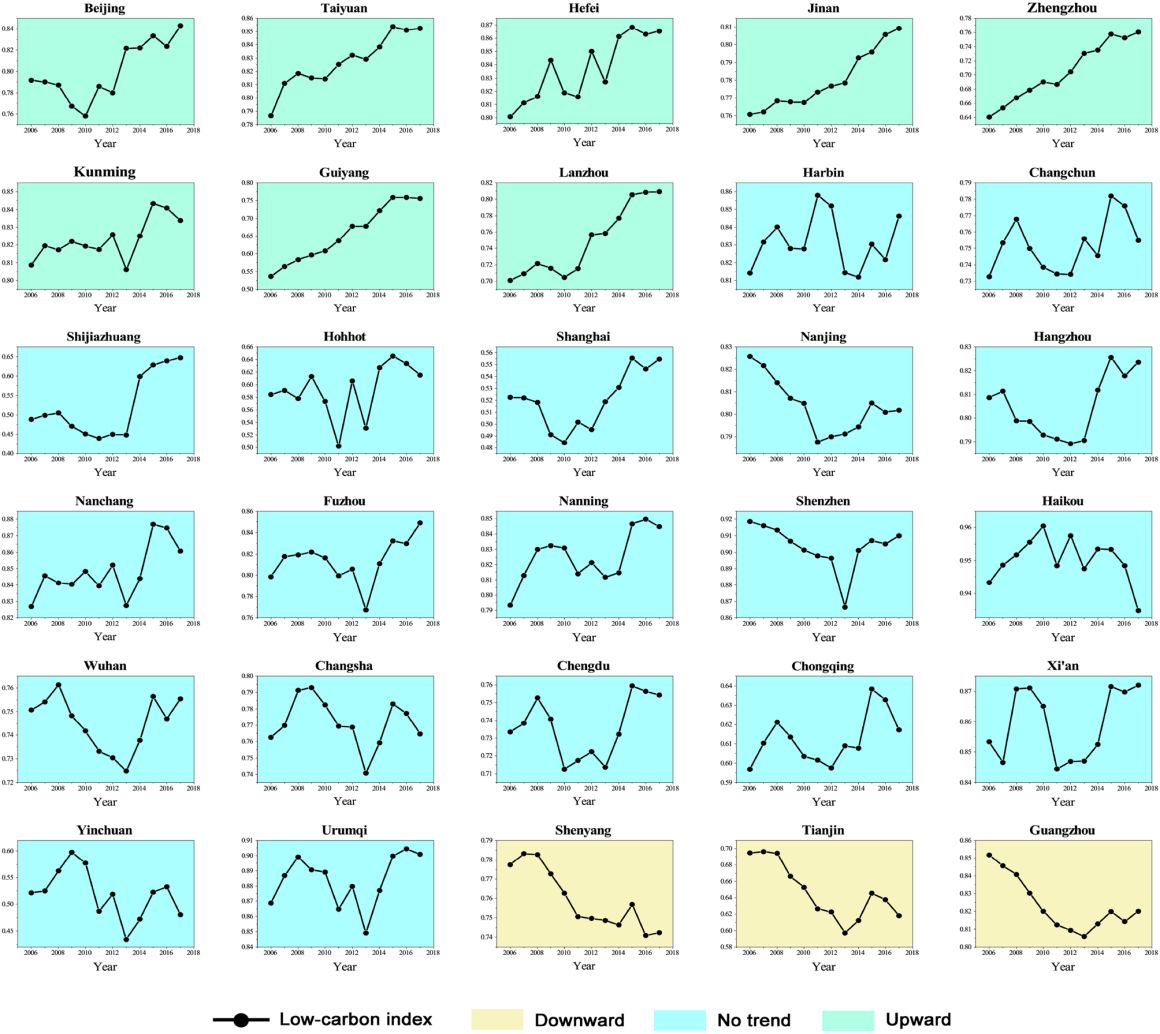
Trend of the low-carbon index in typical cities in China.

In general, the low-carbon index of most cities has shown a clear upward trend over the last 12 years. Figure 3 depicts the results of low-carbon level measurements in typical Chinese cities from 2006 to 2017. Cities with different trends are distinguished by different colors following the Mann-Kendall test. In particular, the low-carbon index of 8 cities has shown a clear upward trend, 19 cities have shown no significant monotonous change trend, and the low-carbon level of 3 cities has dropped significantly, namely Shenyang, Tianjin, and Guangzhou City. Affected by the financial crisis in 2008, global economic growth slowed down, resulting in a sharp drop in external demand. Since before this, Chinese economy was highly dependent on foreign investment, Chinese export economy has been negatively affected to a large extent. To stabilize the growth of the domestic economy, the low-carbon indices of most cities in the study area have declined or stagnated to varying degrees. Therefore, the Chinese government uses financial investment to expand domestic demand, implements a package plan^66^, and uses the multiplier effect and stimulate economic recovery. Among these financial investments, about 37.5% are used for major infrastructure construction such as road network transportation and water conservancy projects^67^.However, many of these industries are carbon-intensive^68^, and some cities have issues with carbon emission efficiency and industrial structure rationalization^69^, which leads to an increase in the overall energy consumption level of cities. The total amount of urban carbon emissions continued to rise, resulting in varying degrees of decline or stagnation in typical city low-carbon indices during this period.

According to the different types of fluctuation trends, for these cities whose low-carbon index is on the rise, the rising trend can be divided into two types, namely: step-up type and oscillating-up type. Among them, the step-up cities include Taiyuan, Jinan, Zhengzhou, Guiyang, and Lanzhou, and the oscillating cities include Beijing, Hefei, and Kunming. For step-up cities, it shows that the industrial transformation effect of such cities is relatively obvious, and the carbon emission efficiency is relatively high, so they are not affected by some carbon-intensive industries during this period. As for the oscillating-up cities, it can be seen that after entering the new normal state of economic development, these cities have been constantly adjusting and optimizing their investment structure, striving to reduce the intensity of urban carbon emissions^70^, making their urban low-carbon index in the There is a certain degree of volatility in the short term, but the long-term upward trend remains unchanged.

For cities with no obvious trend in the MK test, the fluctuation of their low-carbon index can be roughly regarded as a trend of “rising first, then falling, then rising,” that is, the city’s low-carbon index rose slightly before 2008, but there was a relatively obvious decline after 2008. Following a series of new governance concepts and policies such as green innovation and high-quality economic development proposed by the Chinese government^71^, the Low-Carbon City Pilot (LCCP) was implemented^72^ and the Carbon Emissions Trading Scheme (CTS)^73^ and other specific policies, resulting in a significant increase in the city’s low-carbon index after 2012. Furthermore, cities whose low-carbon index change test results show a downward trend must urgently improve their urban carbon emission efficiency. Although such cities’ urban low-carbon index improved from 2013 to 2014, it did not last. Because Tianjin’s average low-carbon index is lower than that of Guangzhou (0.824) and Shenyang (0.759), such cities should make appropriate adjustments to their high-energy-consuming industries, strengthen screening and supervision of foreign investment, and avoid becoming “pollution paradises”^74^.

### Analysis of economic subsystem measurement results

**Figure 4:**
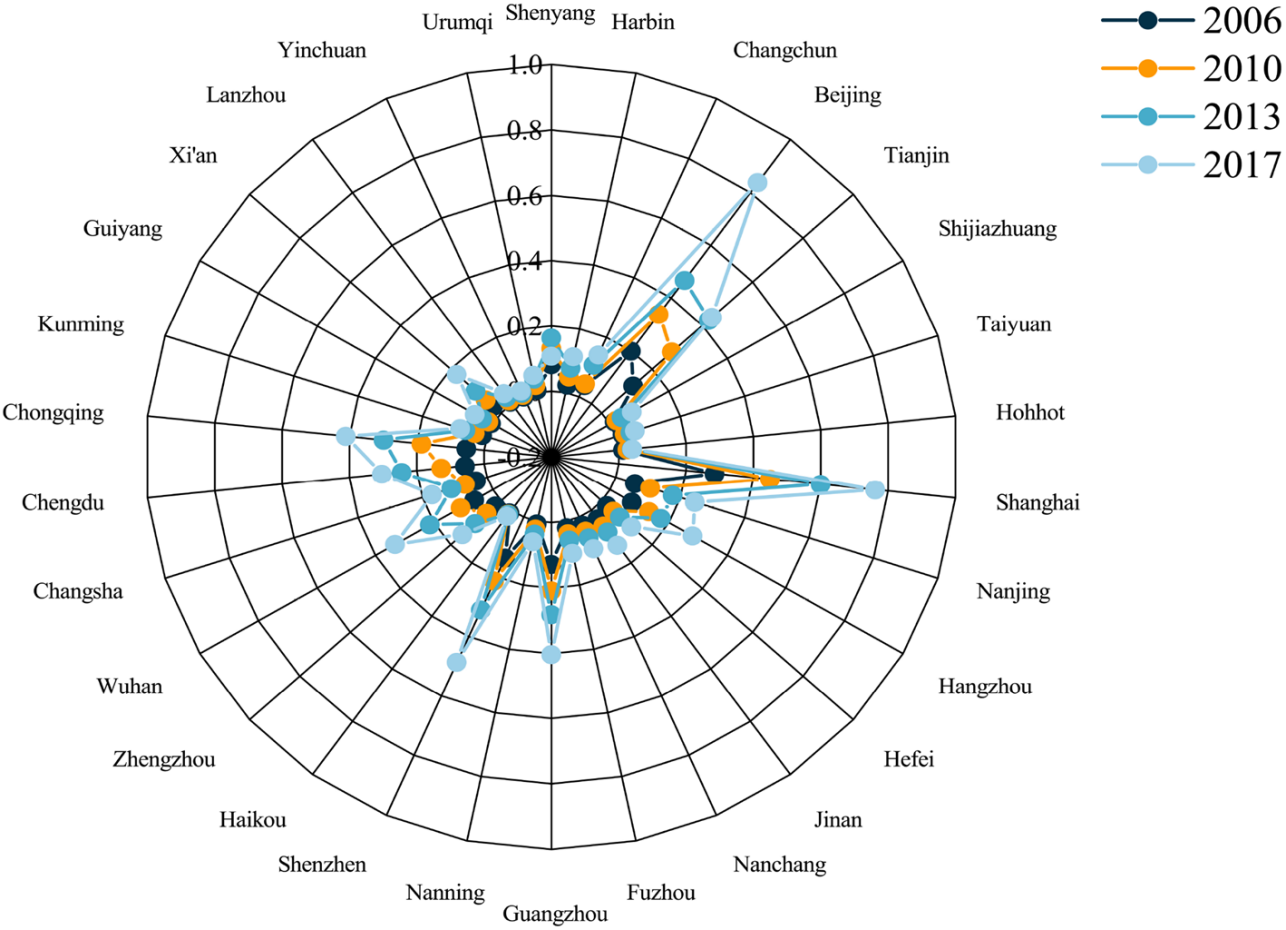
Trend of the economic index in typical cities in China.

Overall, the economic development of Chinese 30 typical cities is improving year after year. As of 2017, the four first-tier cities of “Shanghai, Beijing, Shenzhen, and Guangzhou” maintained strong economic development, while Chengdu, Chongqing, and Wuhan in the inland areas gradually formed new highlands for Chinese urban economic growth (Fig. 4 )

According to the findings in Table 2, the economic development of typical cities across the country has improved significantly between 2006 and 2017. In terms of average economic development, the top five cities are Shanghai (0.533), Beijing (0.436), Tianjin (0.352), Shenzhen (0.282), and Guangzhou (0.254). According to the results, there is no doubt that Shanghai, as Chinese economic center and a window for foreign trade, ranks first in terms of economic level. As the core cities of the Beijing-Tianjin-Hebei urban agglomeration, Beijing and Tianjin are also municipalities directly under the central government’s jurisdiction, and there is a degree of big city siphon effect in the development process. According to economic geography research, the siphoning effect of large cities has both positive and negative externalities. Its positive externality lies in the fact that by absorbing the production factors of the cities in the surrounding areas to flow into the core cities, the development of the core cities will be promoted, and then the development of the surrounding areas will be promoted through the spillover effect, the pressure on resources in the surrounding areas will be relieved, and it will help to improve the ecological environment of the surrounding areas. The negative externality occurs because the core city absorbs a large number of production factors from the surrounding cities, inhibiting their development and forming the core city’s centripetal force^75^, resulting in a relatively high average level of the urban economic index. As far as Guangzhou and Shenzhen are concerned, since China’s accession to the WTO in 2001, due to their geographical advantages and the rapid development of infrastructure such as transportation and road networks, they have become new platforms for China’s opening up to the outside world, enhancing the global differences. The circulation of capital, technology, labor population, knowledge, etc. between cities^76^. In the context of globalization and regional integration, its ability to attract foreign investment has been continuously enhanced, attracting many high-tech professionals and enterprises from all over the world, improving the city’s technological innovation level and total factor productivity, and driving The development of many high value-added and knowledge-intensive industries has promoted the continuous upgrading of the industrial structure^77^, and the urban economy has also been fully developed. In 2016, Chinese “Thirteenth Five-Year Plan” proposed the construction of the Guangdong-Hong Kong-Macao Greater Bay Area, forming a core triangular structure consisting of Guangzhou, Shenzhen, and Hong Kong, seeking new drivers of economic growth through new functional divisions, to further promote the economic development of Guangzhou and Shenzhen, and gradually narrow the gap with Shanghai and Beijing..

In addition, typical cities in Northwest China represented by Lanzhou (0.021), Yinchuan (0.010), and Urumqi (0.033) have average growth rates of 12.4%, 32.3%, and 14.7%, respectively. However, compared with other cities, there is still a certain gap in the absolute value of its economic index. This phenomenon is mainly due to the relatively poor carrying capacity of resources and the environment in western China.The carrying capacity of resources and environment determines the population density of a region to a certain extent, and also determines the adequacy of production factors in a region^78^. As far as Northwest China is concerned, its resource-carrying capacity is relatively weak, resulting in insufficient production factors compared with the eastern coastal areas, so the upper limit of its economic development is lower, which also explains why the economic indexes of these cities are relatively low. There is a good growth rate, but the absolute value level is lower than the reasons behind the results of the cities in the eastern coastal areas.

**Table 2:** Economic subsystem measure results.

City	2006	2007	2008	2009	2010	2011	2012	2013	2014	2015	2016	2017	AVG.
Shenyang	0.082	0.124	0.137	0.129	0.131	0.144	0.156	0.164	0.128	0.118	0.102	0.108	0.127
Harbin	0.025	0.031	0.038	0.043	0.050	0.058	0.069	0.077	0.085	0.099	0.107	0.113	0.066
Changchun	0.039	0.046	0.053	0.038	0.045	0.056	0.092	0.107	0.118	0.127	0.156	0.141	0.085
Beijing	0.200	0.237	0.272	0.300	0.340	0.396	0.435	0.469	0.506	0.595	0.640	0.838	0.436
Tianjin	0.125	0.152	0.193	0.231	0.280	0.335	0.382	0.426	0.472	0.536	0.647	0.439	0.352
Shijiazhuang	0.016	0.017	0.021	0.021	0.023	0.029	0.036	0.041	0.057	0.062	0.069	0.075	0.039
Taiyuan	0.022	0.018	0.022	0.024	0.029	0.036	0.042	0.045	0.049	0.051	0.053	0.060	0.038
Hohhot	0.014	0.017	0.020	0.025	0.026	0.032	0.033	0.039	0.041	0.044	0.048	0.040	0.032
Shanghai	0.284	0.332	0.379	0.405	0.449	0.505	0.555	0.600	0.653	0.707	0.769	0.762	0.533
Nanjing	0.060	0.074	0.084	0.090	0.107	0.132	0.153	0.177	0.185	0.203	0.222	0.245	0.144
Hangzhou	0.075	0.091	0.103	0.117	0.134	0.150	0.162	0.173	0.204	0.231	0.257	0.281	0.165
Hefei	0.021	0.030	0.035	0.034	0.047	0.060	0.064	0.073	0.083	0.093	0.106	0.118	0.064
Jinan	0.033	0.037	0.045	0.050	0.061	0.069	0.074	0.083	0.092	0.099	0.121	0.133	0.075
Nanchang	0.027	0.031	0.034	0.038	0.048	0.055	0.063	0.070	0.077	0.079	0.090	0.106	0.060
Fuzhou	0.020	0.023	0.029	0.033	0.039	0.046	0.052	0.059	0.066	0.073	0.081	0.100	0.052
Guangzhou	0.128	0.147	0.164	0.180	0.208	0.236	0.257	0.282	0.322	0.346	0.370	0.403	0.254
Nanning	0.009	0.012	0.020	0.019	0.024	0.030	0.034	0.040	0.045	0.049	0.054	0.065	0.033
Shenzhen	0.137	0.158	0.177	0.186	0.214	0.251	0.280	0.311	0.346	0.395	0.440	0.486	0.282
Haikou	0.011	0.012	0.012	0.014	0.016	0.012	0.015	0.017	0.018	0.020	0.021	0.023	0.016
Zhengzhou	0.023	0.032	0.040	0.046	0.056	0.081	0.094	0.102	0.115	0.126	0.141	0.153	0.084
Wuhan	0.062	0.071	0.083	0.095	0.110	0.135	0.159	0.215	0.207	0.235	0.267	0.332	0.164
Changsha	0.034	0.041	0.050	0.059	0.068	0.087	0.099	0.111	0.125	0.140	0.155	0.170	0.095
Chengdu	0.056	0.049	0.072	0.085	0.127	0.161	0.200	0.245	0.224	0.229	0.234	0.303	0.166
Chongqing	0.052	0.066	0.097	0.132	0.185	0.274	0.290	0.298	0.328	0.361	0.409	0.411	0.242
Kunming	0.015	0.019	0.025	0.030	0.037	0.047	0.057	0.067	0.074	0.079	0.073	0.083	0.051
Guiyang	0.006	0.007	0.009	0.012	0.015	0.021	0.028	0.035	0.042	0.050	0.055	0.061	0.028
Xi’an	0.030	0.034	0.043	0.049	0.060	0.074	0.086	0.101	0.116	0.130	0.144	0.177	0.087
Lanzhou	0.007	0.008	0.010	0.011	0.013	0.017	0.022	0.025	0.029	0.032	0.036	0.039	0.021
Yinchuan	0.001	0.001	0.003	0.004	0.006	0.010	0.012	0.014	0.016	0.018	0.019	0.021	0.010
Urumqi	0.008	0.012	0.016	0.018	0.023	0.032	0.038	0.043	0.049	0.053	0.050	0.056	0.033

### Analysis of environmental subsystem measurement results

**Figure 5:**
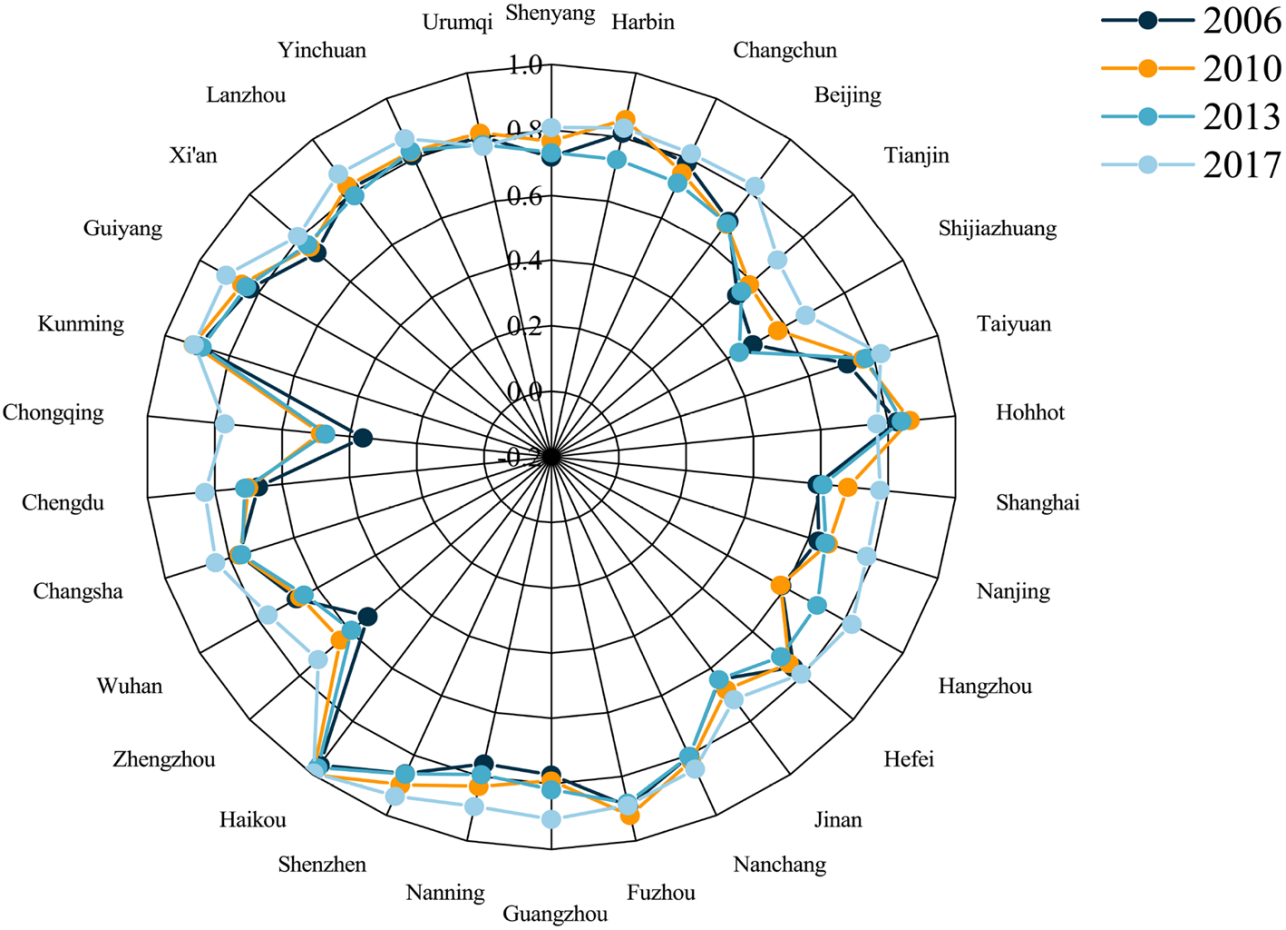
Trend of the environmental index in typical cities in China.

Before 2013, the ecological environment quality of each city did not change significantly. The state of the environment has improved. The environmental conditions of typical southern Chinese coastal cities are generally better than those of other regions, whereas the environmental quality of cities in central China is relatively poor, forming a spatial development trend of “central subsidence” (Fig. 5), for example, Wuhan and Zhengzhou City.The main reason for this result is that the transformation of traditional industries in Central China has not yet been completed, resulting in a relatively low level of rationalization and upgrading of the industrial structure in this region^79^, which leads to the impact of energy-intensive industries on cities. The level of environmental pollution has increased, affecting the city’s overall environmental quality.

According to the results in Table 3, it is found that the typical urban environment across the country has improved to a certain extent between 2006 and 2017. As of 2017, the top five cities with the average environmental index are Haikou (0.978), Kunming (0.886), Shenzhen (0.885), Fuzhou (0.876), and Guiyang (0.853). It shows that the ecological environment of these five cities has significantly improved in the past 15 years. When combined with the evaluation results of the city’s comprehensive level of economic development in Sect. 5.2, Shenzhen’s ranking in both economic and environmental aspects is very high, and this result is also consistent with Shenzhen’s policies and the city’s reasonableness. The planning and the adjustment of the industrial structure are inextricably linked. The Shenzhen Municipal Government worked hard to increase the proportion of tertiary industry in the municipal area from 47.42% in 2006 to 2017.

**Table 3:** environmental subsystem measure results.

City	2006	2007	2008	2009	2010	2011	2012	2013	2014	2015	2016	2017	AVG.
Shenyang	0.718	0.721	0.703	0.750	0.766	0.728	0.754	0.731	0.654	0.678	0.795	0.807	0.734
Harbin	0.813	0.826	0.805	0.852	0.855	0.780	0.798	0.731	0.671	0.757	0.856	0.829	0.798
Changchun	0.784	0.772	0.756	0.797	0.750	0.673	0.768	0.718	0.710	0.692	0.837	0.817	0.756
Beijing	0.691	0.696	0.709	0.689	0.679	0.678	0.708	0.683	0.687	0.733	0.775	0.824	0.713
Tianjin	0.538	0.551	0.565	0.569	0.588	0.560	0.592	0.555	0.506	0.595	0.650	0.700	0.581
Shijiazhuang	0.487	0.499	0.582	0.590	0.573	0.489	0.491	0.441	0.466	0.563	0.610	0.667	0.538
Taiyuan	0.720	0.749	0.779	0.796	0.768	0.711	0.765	0.778	0.774	0.817	0.836	0.823	0.776
Hohhot	0.827	0.881	0.882	0.876	0.865	0.848	0.863	0.841	0.812	0.862	0.816	0.767	0.845
Shanghai	0.591	0.591	0.618	0.641	0.681	0.639	0.655	0.607	0.568	0.590	0.696	0.776	0.638
Nanjing	0.630	0.626	0.634	0.647	0.660	0.682	0.713	0.652	0.608	0.659	0.742	0.781	0.670
Hangzhou	0.585	0.578	0.576	0.571	0.582	0.669	0.710	0.707	0.676	0.734	0.801	0.825	0.668
Hefei	0.761	0.731	0.741	0.737	0.744	0.728	0.755	0.714	0.639	0.705	0.798	0.793	0.737
Jinan	0.642	0.654	0.673	0.669	0.681	0.606	0.645	0.642	0.657	0.594	0.670	0.718	0.654
Nanchang	0.802	0.794	0.797	0.810	0.817	0.779	0.804	0.802	0.800	0.842	0.835	0.845	0.811
Fuzhou	0.892	0.896	0.901	0.911	0.921	0.880	0.887	0.882	0.781	0.811	0.858	0.890	0.876
Guangzhou	0.772	0.773	0.775	0.771	0.790	0.769	0.797	0.818	0.848	0.896	0.918	0.908	0.820
Nanning	0.759	0.774	0.777	0.829	0.830	0.787	0.795	0.793	0.819	0.859	0.903	0.893	0.818
Shenzhen	0.860	0.852	0.859	0.865	0.898	0.848	0.861	0.862	0.899	0.931	0.946	0.936	0.885
Haikou	0.964	0.959	0.949	0.983	1.000	0.946	0.994	0.975	0.984	0.993	0.997	0.993	0.978
Zhengzhou	0.530	0.549	0.620	0.632	0.638	0.549	0.588	0.594	0.612	0.587	0.701	0.727	0.611
Wuhan	0.670	0.658	0.642	0.654	0.658	0.634	0.680	0.644	0.660	0.700	0.747	0.767	0.676
Changsha	0.769	0.766	0.735	0.761	0.768	0.746	0.757	0.763	0.751	0.811	0.846	0.843	0.776
Chengdu	0.670	0.695	0.686	0.686	0.700	0.736	0.747	0.710	0.775	0.802	0.803	0.829	0.737
Chongqing	0.359	0.406	0.418	0.430	0.485	0.455	0.479	0.471	0.461	0.496	0.710	0.770	0.495
Kunming	0.897	0.901	0.903	0.914	0.912	0.727	0.845	0.886	0.906	0.920	0.916	0.910	0.886
Guiyang	0.828	0.838	0.838	0.859	0.856	0.839	0.840	0.840	0.825	0.873	0.891	0.910	0.853
Xi’an	0.732	0.737	0.770	0.771	0.758	0.677	0.738	0.769	0.801	0.836	0.802	0.808	0.767
Lanzhou	0.812	0.820	0.823	0.829	0.826	0.783	0.805	0.789	0.763	0.817	0.864	0.870	0.817
Yinchuan	0.809	0.835	0.854	0.860	0.821	0.809	0.809	0.824	0.843	0.843	0.857	0.866	0.836
Urumqi	0.800	0.794	0.802	0.804	0.813	0.761	0.769	0.777	0.766	0.807	0.781	0.772	0.787

### Analysis of coupling coordination degree results

To further explore the impact mechanism among urban low-carbon level, urban economic development, and urban ecological environment, this research establishes a low-carbon-economic-environment multi-coupling coordination model for 30 typical cities. By measuring the low-carbon-economic-environment index coupling coordination degree, low-carbon-economic index coupling coordination degree, and low-carbon-environment index coupling coordination degree, and referring to previous studies^80^, the coupling coordination degree is divided into five The intervals are poor coordination (0 $\le$ D $\le$ 0.2), weak coordination (0.2$\le$ D$\le$0.4), basic coordination (0.4 $\le$ D $\le$ 0.6), good coordination (0.6 $\le$ D $\le$ 0.8), excellent coordination (0.8 $\le$ D $\le$ 1.0), to conduct in-depth exploration and analysis on the mechanism of urban low-carbon level impact.

**Figure 6:**
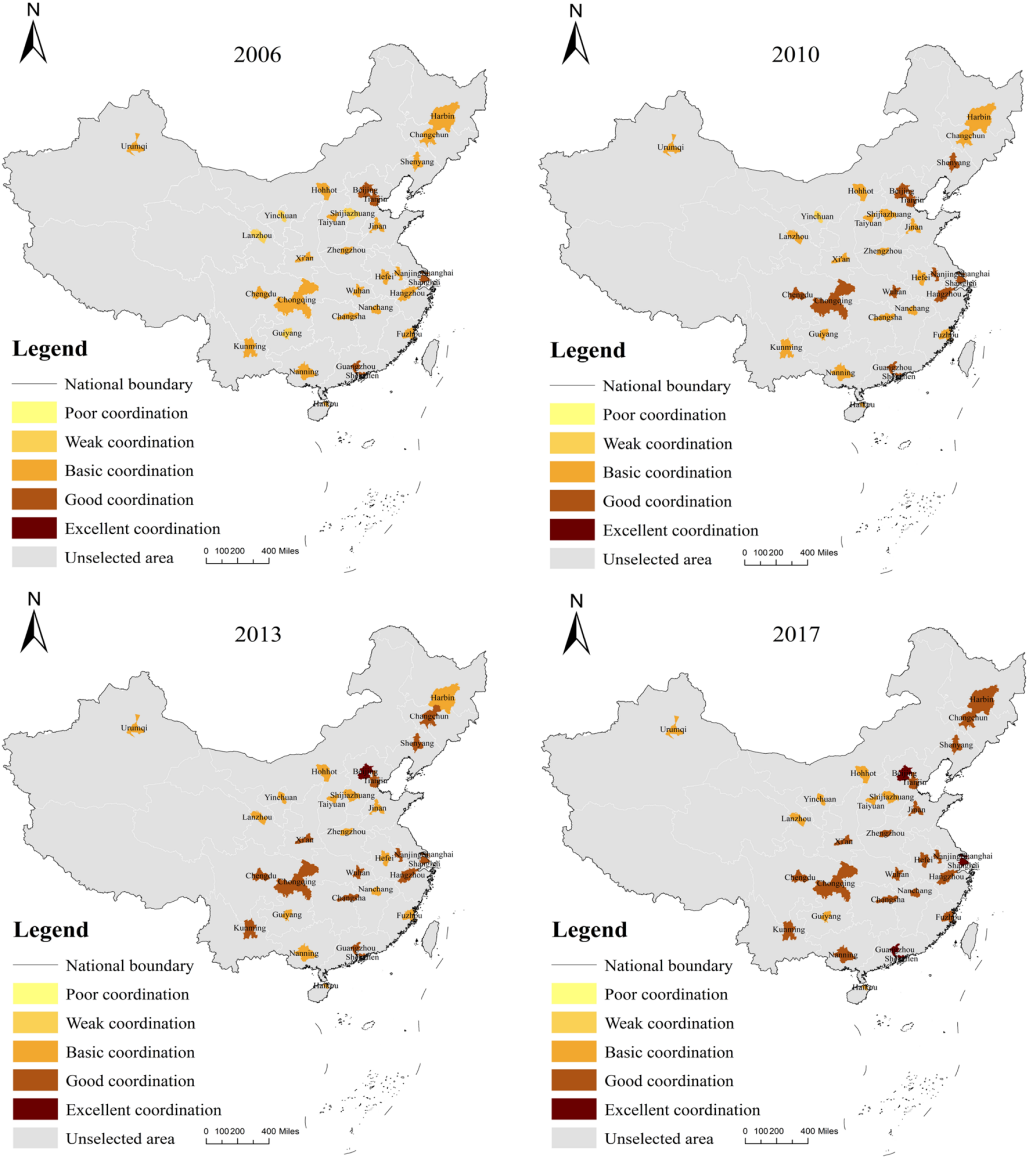
CCD of low-carbon-economic-environment index.

Except for Haikou City, most cities’ low-carbon-economic-environment index coupling coordination shows an upward trend at different levels. Table 4 shows the calculation results of the low-carbon-economic-environment index coupling coordination degree of 30 typical cities in China from 2006 to 2017. From the standpoint of spatial change characteristics, cities with high coupling and coordination of low-carbon-economic-environment indexes are primarily concentrated in four urban agglomerations, namely the Beijing-Tianjin-Hebei urban agglomeration, the Yangtze River Delta urban agglomeration, the Pearl River Delta urban agglomeration, and the Chengdu-Chongqing urban agglomeration (see Fig. 6). In terms of time characteristics, only the typical city coupling coordination degree of the Beijing-Tianjin-Hebei urban agglomeration, Yangtze River Delta urban agglomeration, and Pearl River Delta urban agglomeration were at the stage of good coupling in 2006, while the remaining regions were essentially between weak coupling and basic coupling. By 2010, the coupling coordination between Chengdu and Chongqing had reached a positive coupling, indicating that the economic development of Chengdu and Chongqing has gradually decreased its reliance on carbon-intensive industries, and the economic focus has gradually shifted to the tertiary industry, primarily the service industry, resulting in a decrease in total emissions of carbon dioxide and other industrial pollutants in the cities. As a result, the urban environment has significantly improved.In addition, from the results of coupling coordination in 2013 and 2017, the Beijing-Tianjin-Hebei urban agglomeration has driven the coordinated development of typical cities in the northeast region to the north, while the Yangtze River Delta urban agglomeration and the Chengdu-Chongqing urban agglomeration have positively influenced the typical cities in central China, which are in the middle of the Yangtze River economic belt, through the Yangtze River. And the Pearl River Delta region is driving the coordinated development of Guangxi province.The main reason for this phenomenon is that, with the change of the Chinese government’s goal of economic growth from high growth rate to high quality, some industries with high energy consumption and low efficiency have been eliminated one after another, and the transformation and upgrading of industries through technological innovation have changed the industrial structure, optimized the allocation and efficiency of factor markets, and produced technological spillover effects on cities in the surrounding areas while developing itself, driving the technological progress and industrial structure upgrading in other surrounding areas, thus also making the coupling and coordination of the low-carbon-economic-environment multiple systems in the surrounding areas tend to be good .

**Figure 7:**
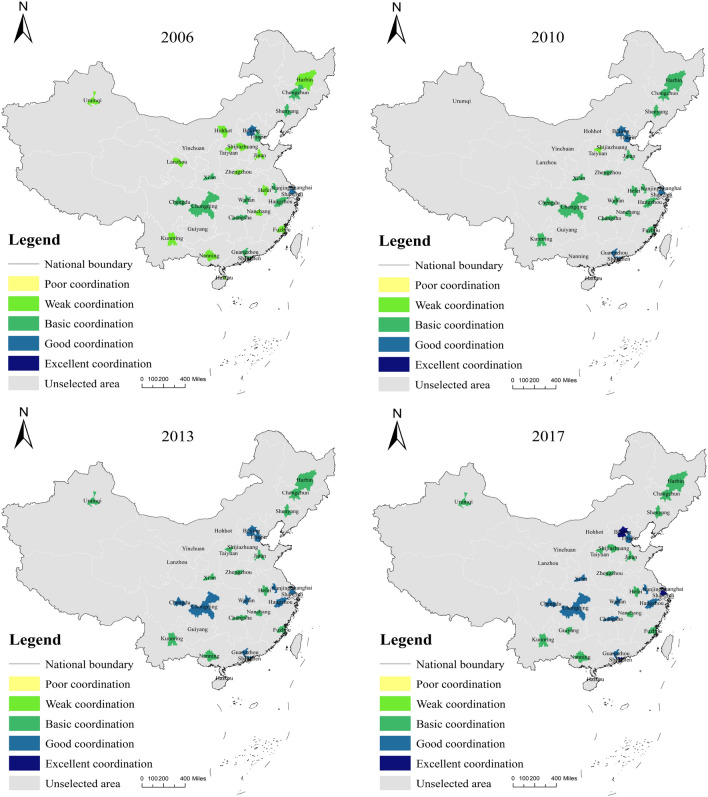
CCD of low-carbon-economic index.

Based on the results of the coupling coordination degree, combined with the evaluation results of the low-carbon subsystem and economic subsystem, this research makes a judgment on the role mechanism between urban carbon emission and economic development. On the one hand, the southeast region is generally better than the northwest region in terms of spatial distribution, and this result is primarily because the economies of most cities in the southeast region are better than those of the northwest region. On the other hand, the economic development of coastal cities in East China has gradually shifted away from the dependence on energy-consuming industries, which is because many high-tech industries have gradually moved to the coastal cities in East China, and these enterprises are characterized by high output value, low pollution, and high technological innovation capacity, etc. More and more urban real enterprises will transform in this direction in the future, which is an inevitable trend (Fig. 7). As for the three cities in Northeast China, they still rely on the heavy industrial development of the old Northeast industrial zone in the past 15 years, and the coupling coordination of their low-carbon-economic indexes has been at the basic coupling level, which needs to be highly emphasized by the authorities concerned in this regard. The industrial structure of the Northeast has been one of the core issues of concern and discussion in academia and society, and its internal relationships are intricate and complex. In terms of results alone, the core driver of economic development in the Northeast is still a carbon-intensive heavy industry, and it will take some time to achieve high-quality development. It is worth noting that in the early part of this century, Chinese economic growth model relied heavily on investment, and local governments often used land finance to attract investment to further increase economic growth, thus investing large amounts of capital in infrastructure construction, creating a “multiplier effect” that, although it had a significant short-term impact on Chinese economic Although this had a significant impact on Chinese economic growth rate in the short term, the infrastructure boom exacerbated overcapacity and led to the so-called “sloppy” economic growth, which led to the coordination of economic development and low levels of urban carbon emissions in most Chinese cities during that period. After the 12th Five-Year Plan, Chinese economic development began to shift gears and speed, eliminating backward production capacity, gradually focusing on consumption-driven economic development, and further optimizing the industrial structure, which led to a gradual shift of the low-carbon-economic index coupling coordination to a high level of coordination in most cities from 2013 onwards (Table 5 ).

**Figure 8:**
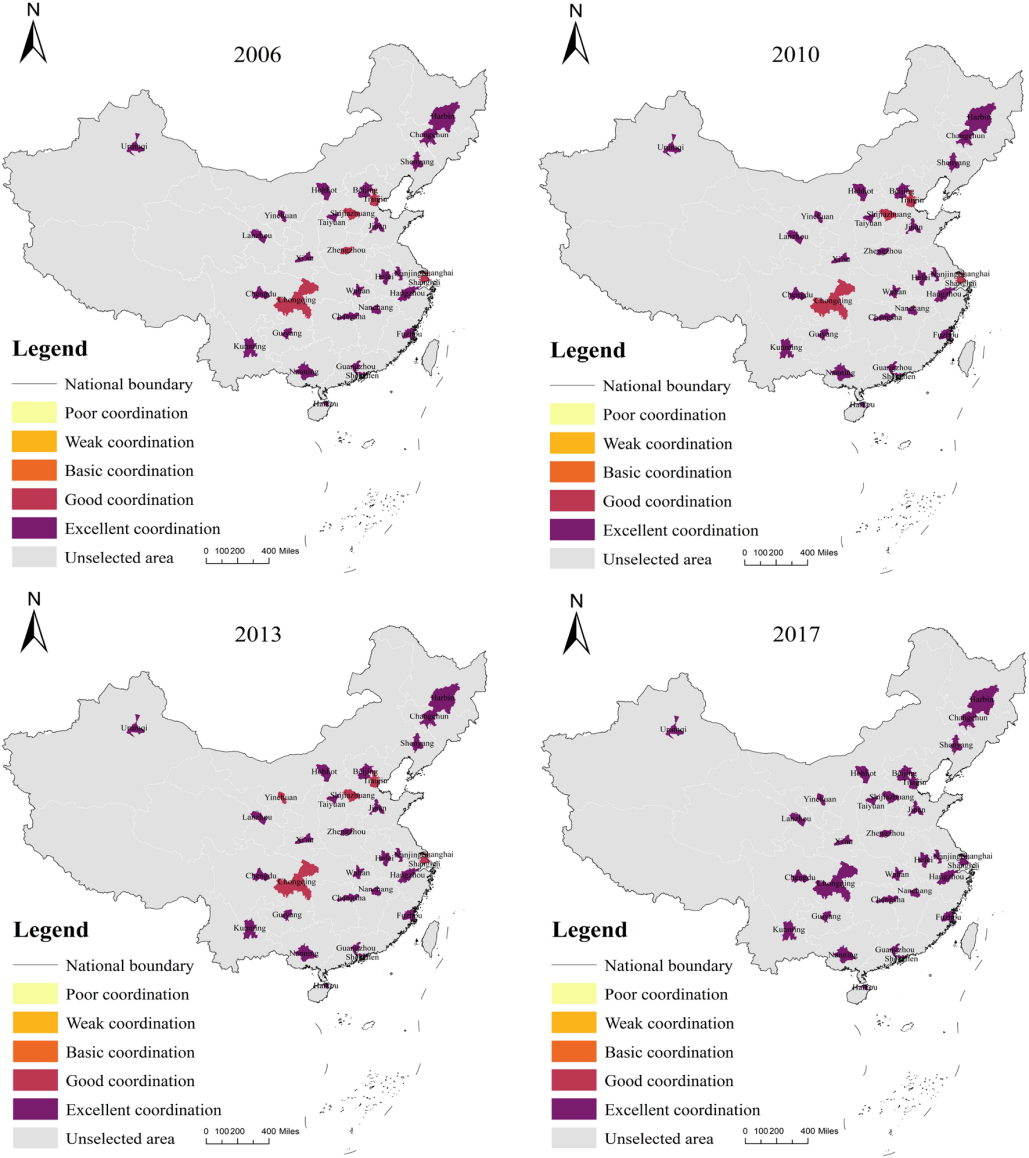
CCD of low-carbon-environment index.

Furthermore, the improvement of urban low-carbon levels cannot be separated from the management of the urban ecological environment; urban low-carbon development can promote the improvement of the urban ecological environment, while the quality improvement of the ecological environment will counteract the urban low-carbon level, and the two affect and interact with each other. Most cities’ average low-carbon-environment index coupling coordination level is generally high (Fig. 8). The average level of each city is calculated based on the results in Table 6, and it is discovered that Haikou (0.982) and Shenzhen (0.945) have the highest coupling coordination level of low-carbon-environment index, indicating that the ecological environment of these two cities is at a better level while achieving low-carbon development in the cities. During the 2013–2015 period, Shenzhen actively responded to the national 12th Five-Year Plan by releasing activities such as “Pengcheng Waste Reduction,” optimizing and adjusting the industrial structure, vigorously developing a clean energy structure strategy, and gradually guiding the change of market investment direction. The market investment direction has shifted so that the city of Shenzhen can ensure effective carbon emission control while also considering enterprise development. In Haikou, forested areas account for approximately 42% of the total land area, the tertiary industry is dominant, and there are few large heavy industrial enterprises. For a long time, Haikou has had a high level of coupling coordination of low-carbon and environmental indices.

**Table 4:** CCD of low-carbon-economic-environment index from 2006 to 2017.

City	2006	2007	2008	2009	2010	2011	2012	2013	2014	2015	2016	2017	AVG.
Shenyang	0.598	0.642	0.650	0.649	0.652	0.655	0.667	0.669	0.630	0.626	0.626	0.633	0.641
Harbin	0.504	0.528	0.544	0.558	0.573	0.582	0.601	0.599	0.599	0.629	0.649	0.656	0.585
Changchun	0.531	0.547	0.560	0.533	0.540	0.550	0.611	0.623	0.630	0.640	0.683	0.666	0.593
Beijing	0.692	0.712	0.730	0.736	0.748	0.772	0.788	0.801	0.812	0.845	0.861	0.914	0.784
Tianjin	0.601	0.623	0.651	0.666	0.689	0.700	0.721	0.722	0.726	0.768	0.803	0.758	0.702
Shijiazhuang	0.397	0.400	0.427	0.425	0.425	0.430	0.447	0.448	0.502	0.529	0.548	0.565	0.462
Taiyuan	0.480	0.473	0.492	0.501	0.511	0.526	0.546	0.555	0.562	0.574	0.579	0.589	0.532
Hohhot	0.433	0.455	0.466	0.487	0.485	0.488	0.509	0.508	0.525	0.539	0.541	0.517	0.496
Shanghai	0.667	0.684	0.704	0.710	0.727	0.738	0.751	0.757	0.763	0.784	0.815	0.831	0.744
Nanjing	0.561	0.580	0.593	0.601	0.620	0.643	0.665	0.671	0.669	0.690	0.713	0.732	0.645
Hangzhou	0.574	0.591	0.601	0.613	0.629	0.656	0.671	0.678	0.694	0.720	0.743	0.759	0.661
Hefei	0.483	0.510	0.526	0.525	0.554	0.573	0.588	0.592	0.597	0.620	0.646	0.658	0.573
Jinan	0.502	0.513	0.534	0.543	0.562	0.565	0.578	0.588	0.602	0.601	0.635	0.652	0.573
Nanchang	0.513	0.526	0.533	0.544	0.568	0.574	0.592	0.600	0.611	0.623	0.635	0.653	0.581
Fuzhou	0.494	0.507	0.527	0.539	0.555	0.565	0.578	0.584	0.589	0.605	0.621	0.650	0.568
Guangzhou	0.662	0.677	0.689	0.698	0.716	0.727	0.741	0.755	0.778	0.796	0.807	0.818	0.739
Nanning	0.416	0.440	0.483	0.486	0.505	0.517	0.530	0.544	0.557	0.574	0.588	0.605	0.520
Shenzhen	0.690	0.706	0.720	0.726	0.747	0.759	0.775	0.784	0.809	0.833	0.850	0.863	0.772
Haikou	0.462	0.470	0.471	0.486	0.499	0.472	0.490	0.499	0.509	0.515	0.520	0.529	0.493
Zhengzhou	0.445	0.474	0.505	0.520	0.540	0.559	0.582	0.595	0.610	0.619	0.648	0.662	0.563
Wuhan	0.561	0.573	0.586	0.599	0.614	0.630	0.655	0.681	0.682	0.706	0.728	0.760	0.648
Changsha	0.520	0.539	0.555	0.574	0.587	0.607	0.622	0.630	0.644	0.668	0.683	0.692	0.610
Chengdu	0.549	0.541	0.578	0.592	0.631	0.663	0.690	0.706	0.709	0.720	0.722	0.758	0.655
Chongqing	0.472	0.504	0.542	0.571	0.615	0.650	0.661	0.664	0.672	0.697	0.754	0.762	0.630
Kunming	0.472	0.492	0.513	0.530	0.551	0.550	0.585	0.603	0.618	0.628	0.619	0.631	0.566
Guiyang	0.368	0.390	0.408	0.426	0.444	0.474	0.503	0.521	0.541	0.568	0.577	0.590	0.484
Xi’an	0.517	0.525	0.553	0.565	0.584	0.590	0.614	0.636	0.655	0.675	0.682	0.707	0.609
Lanzhou	0.394	0.411	0.423	0.433	0.442	0.461	0.488	0.497	0.508	0.524	0.540	0.550	0.473
Yinchuan	0.257	0.288	0.326	0.360	0.379	0.397	0.412	0.414	0.429	0.445	0.452	0.453	0.384
Urumqi	0.424	0.450	0.476	0.483	0.506	0.526	0.544	0.553	0.567	0.581	0.574	0.582	0.522

**Table 5:** CCD of low-carbon-economic index from 2006 to 2017.

City	2006	2007	2008	2009	2010	2011	2012	2013	2014	2015	2016	2017	AVG.
Shenyang	0.503	0.558	0.573	0.562	0.562	0.574	0.585	0.592	0.556	0.546	0.524	0.532	0.555
Harbin	0.377	0.402	0.424	0.434	0.451	0.473	0.493	0.501	0.512	0.535	0.544	0.556	0.475
Changchun	0.411	0.431	0.449	0.411	0.426	0.450	0.510	0.534	0.544	0.561	0.590	0.571	0.491
Beijing	0.631	0.658	0.680	0.693	0.712	0.747	0.763	0.788	0.803	0.839	0.852	0.917	0.757
Tianjin	0.543	0.570	0.605	0.627	0.654	0.677	0.698	0.710	0.733	0.767	0.802	0.722	0.676
Shijiazhuang	0.300	0.301	0.320	0.316	0.318	0.337	0.357	0.368	0.430	0.444	0.458	0.470	0.368
Taiyuan	0.361	0.349	0.367	0.376	0.391	0.416	0.432	0.441	0.449	0.457	0.461	0.475	0.415
Hohhot	0.299	0.317	0.328	0.351	0.350	0.356	0.377	0.379	0.401	0.411	0.419	0.397	0.365
Shanghai	0.621	0.645	0.666	0.668	0.683	0.710	0.724	0.747	0.767	0.792	0.805	0.806	0.720
Nanjing	0.472	0.496	0.511	0.520	0.542	0.568	0.589	0.612	0.620	0.636	0.649	0.666	0.573
Hangzhou	0.497	0.521	0.535	0.553	0.571	0.587	0.598	0.608	0.638	0.661	0.677	0.694	0.595
Hefei	0.359	0.394	0.411	0.411	0.444	0.470	0.484	0.496	0.516	0.533	0.549	0.565	0.469
Jinan	0.397	0.409	0.430	0.443	0.464	0.481	0.490	0.504	0.519	0.530	0.559	0.572	0.483
Nanchang	0.388	0.404	0.412	0.423	0.450	0.463	0.482	0.491	0.505	0.513	0.529	0.550	0.467
Fuzhou	0.357	0.371	0.393	0.405	0.423	0.438	0.453	0.461	0.481	0.496	0.509	0.540	0.444
Guangzhou	0.575	0.593	0.610	0.622	0.643	0.662	0.675	0.690	0.715	0.730	0.741	0.758	0.668
Nanning	0.287	0.311	0.357	0.355	0.376	0.394	0.409	0.425	0.437	0.452	0.462	0.484	0.396
Shenzhen	0.595	0.617	0.634	0.641	0.663	0.689	0.708	0.720	0.747	0.774	0.794	0.816	0.700
Haikou	0.317	0.325	0.328	0.340	0.352	0.329	0.343	0.354	0.364	0.371	0.375	0.385	0.349
Zhengzhou	0.348	0.379	0.404	0.421	0.444	0.486	0.507	0.523	0.539	0.556	0.570	0.584	0.480
Wuhan	0.465	0.481	0.501	0.516	0.534	0.561	0.584	0.628	0.625	0.649	0.668	0.708	0.577
Changsha	0.401	0.423	0.447	0.466	0.481	0.509	0.525	0.535	0.555	0.575	0.589	0.600	0.509
Chengdu	0.450	0.436	0.482	0.501	0.549	0.583	0.617	0.647	0.637	0.646	0.649	0.692	0.574
Chongqing	0.419	0.449	0.496	0.534	0.578	0.637	0.645	0.653	0.668	0.693	0.713	0.710	0.600
Kunming	0.333	0.354	0.377	0.395	0.418	0.442	0.467	0.482	0.497	0.508	0.498	0.513	0.440
Guiyang	0.234	0.255	0.273	0.289	0.308	0.341	0.372	0.392	0.418	0.442	0.451	0.464	0.353
Xi’an	0.402	0.410	0.439	0.453	0.478	0.500	0.519	0.541	0.560	0.580	0.595	0.627	0.509
Lanzhou	0.261	0.277	0.288	0.299	0.308	0.332	0.359	0.372	0.387	0.399	0.412	0.422	0.343
Yinchuan	0.137	0.162	0.194	0.224	0.245	0.263	0.279	0.280	0.293	0.310	0.315	0.316	0.252
Urumqi	0.291	0.319	0.347	0.355	0.379	0.409	0.428	0.438	0.456	0.468	0.462	0.473	0.402

**Table 6:** CCD of low-carbon-environment index from 2006 to 2017.

City	2006	2007	2008	2009	2010	2011	2012	2013	2014	2015	2016	2017	AVG.
Shenyang	0.864	0.867	0.861	0.872	0.874	0.860	0.867	0.860	0.836	0.846	0.876	0.880	0.864
Harbin	0.902	0.910	0.907	0.917	0.917	0.904	0.908	0.878	0.859	0.890	0.916	0.915	0.902
Changchun	0.870	0.873	0.873	0.879	0.863	0.839	0.866	0.858	0.853	0.858	0.898	0.886	0.868
Beijing	0.860	0.861	0.865	0.853	0.847	0.855	0.862	0.865	0.867	0.884	0.894	0.913	0.869
Tianjin	0.782	0.787	0.792	0.785	0.787	0.770	0.779	0.759	0.746	0.787	0.802	0.811	0.782
Shijiazhuang	0.698	0.707	0.736	0.726	0.713	0.681	0.685	0.667	0.727	0.771	0.790	0.810	0.726
Taiyuan	0.868	0.883	0.893	0.898	0.889	0.875	0.893	0.896	0.897	0.914	0.918	0.915	0.895
Hohhot	0.834	0.849	0.845	0.856	0.839	0.808	0.850	0.817	0.845	0.864	0.848	0.829	0.840
Shanghai	0.745	0.745	0.752	0.749	0.758	0.752	0.755	0.749	0.741	0.757	0.785	0.810	0.758
Nanjing	0.849	0.847	0.848	0.850	0.854	0.856	0.866	0.847	0.834	0.853	0.878	0.889	0.856
Hangzhou	0.829	0.827	0.824	0.822	0.824	0.853	0.865	0.865	0.861	0.882	0.900	0.908	0.855
Hefei	0.884	0.878	0.882	0.888	0.884	0.878	0.895	0.877	0.861	0.884	0.911	0.910	0.886
Jinan	0.836	0.840	0.848	0.847	0.850	0.827	0.841	0.841	0.850	0.829	0.857	0.873	0.845
Nanchang	0.903	0.905	0.905	0.908	0.912	0.899	0.910	0.902	0.907	0.927	0.924	0.924	0.911
Fuzhou	0.919	0.925	0.927	0.930	0.931	0.916	0.919	0.907	0.892	0.906	0.918	0.932	0.919
Guangzhou	0.901	0.899	0.899	0.894	0.897	0.889	0.896	0.901	0.911	0.926	0.930	0.929	0.906
Nanning	0.881	0.890	0.896	0.911	0.911	0.895	0.899	0.896	0.904	0.923	0.936	0.932	0.906
Shenzhen	0.943	0.940	0.941	0.941	0.948	0.934	0.937	0.930	0.949	0.959	0.962	0.961	0.945
Haikou	0.977	0.977	0.975	0.985	0.990	0.973	0.988	0.980	0.984	0.986	0.986	0.982	0.982
Zhengzhou	0.763	0.774	0.802	0.809	0.815	0.784	0.802	0.811	0.819	0.817	0.852	0.862	0.809
Wuhan	0.842	0.839	0.836	0.836	0.836	0.826	0.840	0.826	0.835	0.853	0.864	0.872	0.842
Changsha	0.875	0.876	0.873	0.881	0.880	0.870	0.873	0.867	0.869	0.893	0.900	0.896	0.880
Chengdu	0.837	0.846	0.848	0.844	0.840	0.852	0.857	0.844	0.868	0.884	0.883	0.889	0.858
Chongqing	0.681	0.705	0.714	0.717	0.735	0.723	0.731	0.732	0.728	0.750	0.819	0.830	0.739
Kunming	0.923	0.927	0.927	0.931	0.930	0.878	0.914	0.919	0.930	0.939	0.937	0.933	0.924
Guiyang	0.816	0.829	0.836	0.846	0.849	0.855	0.868	0.869	0.878	0.902	0.907	0.911	0.864
Xi’an	0.889	0.889	0.905	0.905	0.900	0.870	0.889	0.898	0.909	0.924	0.914	0.916	0.901
Lanzhou	0.869	0.873	0.878	0.878	0.873	0.865	0.883	0.879	0.878	0.901	0.914	0.916	0.884
Yinchuan	0.806	0.814	0.832	0.847	0.830	0.792	0.805	0.773	0.795	0.815	0.822	0.803	0.811
Urumqi	0.913	0.916	0.922	0.920	0.922	0.901	0.907	0.901	0.905	0.923	0.917	0.913	0.913

### Analysis of core driver results

**Figure 9:**
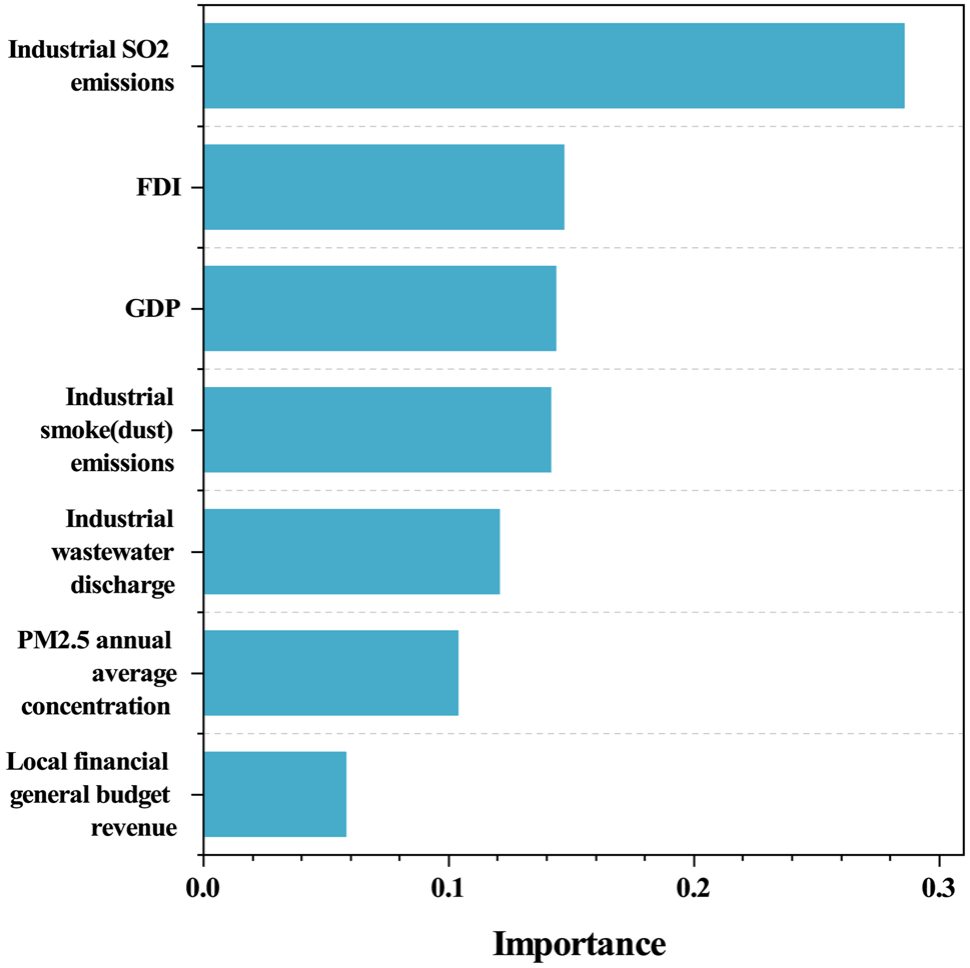
Importance of indicators.

Total industrial sulfur dioxide emissions, foreign direct investment (FDI), and gross regional product (GDP) are the three factors with the greatest influence on the low-carbon level of cities in the economic and environmental subsystems, respectively, according to the overall results, with total industrial sulfur dioxide emissions being more important to total urban carbon emissions than the other two factors, indicating a strong correlation between the two (Fig. 9). On the one hand, industrial sulfur dioxide, a major air pollutant, is produced by the industrial sector^81^, particularly by some energy-intensive secondary industries; on the other hand, due to factor endowments, the current energy consumption structure in China is still dominated by traditional fossil energy. However, due to factor endowments, the current energy consumption structure in China is still dominated by traditional fossil energy, and traditional fossil energy fuels are the main contributors to total urban CO$_2$ emissions^82^, so energy-consuming intensive industries are also one of the important sources of urban CO$_2$ emissions. In summary, traditional energy-intensive industries emit carbon dioxide into the atmosphere as well as sulfur dioxide gas during the mass production process, so there is a strong direct correlation between the two. For urban environmental conditions, an increase in total emissions of air pollutants such as sulfur dioxide will have a direct negative impact on the ecological quality of cities^83^, so the proportion of energy-intensive industries should be appropriately adjusted in tandem with economic development, and environmental regulations should be strengthened to help reduce pollutant emissions and thus improve ecological quality. Furthermore, foreign direct investment and regional economic output level are the primary drivers of total urban CO$_2$ emissions, indicating that foreign investment and regional economic output level have a significant impact on cities’ low-carbon levels (the details will be discussed in the next section).

## Discussion

According to the calculation results of the coupling coordination between the low-carbon level and the economic development level of 30 typical cities in China, as of 2017, the coupling and coordination level of the typical cities along the Yangtze River Economic Belt and the core cities of major urban agglomerations tends to be good. Most typical cities’ low-carbon-economic coupling coordination level has increased since 2006. Due to the “extensive” development of the national economy in 2006, the low-carbon level of most typical cities in the country was still in conflict with the economy, and economic development would impede the city’s low-carbon level’s rise. But in the follow-up, with the transformation of national policies and the development of the urban economy to a certain scale, the low-carbon level and economic development of these typical cities gradually tend to be in good harmony, indicating that both are growing at the same high level. The relationship between the two shows a trend of rising first and then falling, which to some extent supports the “inverted U-shaped” relationship proposed by many scholars and the environmental Kuznets curve hypothesis^84,85^.

The specific reasons for this phenomenon, in addition to the theoretical basis provided by the EKC hypothesis, this research explains the inverted U-shaped relationship between the economy and carbon emissions of typical Chinese cities based on the “promotion tournament” theory. From the perspective of Chinese economy and government system, objectively, compared with other industries, carbon emission-intensive industries can rapidly increase regional economic output in a short period; subjectively, some local officials may be affected by “ Influenced by the “promoting tournament governance model”^86,87^, setting higher economic growth indicators^88^, and investing a large number of production materials in industries that are used to increase the speed of economic development, so in terms of urban construction and economic development, The high degree of dependence on carbon emission-intensive industries will lead to an increase in the city’s total carbon emissions and reduce the city’s low-carbon level while the economic level is growing. In the later period, with the further expansion of the level of economic development and the government’s macro-control, the growth target of the local economy has shifted from high growth to high quality, which has eased the local enthusiasm for economic growth to a certain extent, and through technological innovation and other means Accelerate industrial innovation, eliminate outdated industries with high energy consumption and low production efficiency, optimize industrial structure and factor allocation methods, and improve the distribution efficiency of production materials, to gradually decouple economic growth from carbon emissions, and finally achieve a low-carbon-economic benign develop. It should be noted that the “promotion tournament” theory, the hypothesis that there is a relationship between economic growth and promotion, is currently controversial in Chinese academic circles, and some empirical studies have shown that there is no significant relationship between the two^89,90^.

Furthermore, foreign direct investment is an important factor for typical cities in other regions of China, except for the western region, in the result analysis of the core driving factors of low-carbon levels in typical cities in China. As can be seen, foreign investment plays a significant role in improving the low-carbon level of typical Chinese cities. Foreign direct investment’s impact on carbon emissions has long been a hot topic in international academic circles. It is widely assumed that the impact of foreign direct investment on the host country’s environment has two sides. On the positive side, according to the pollution halo hypothesis^91^, foreign direct investment can promote the improvement of local green total factor productivity through the host country’s spillover effect of technology and knowledge^92^, thereby assisting the host country in improving its ecological environment. On the negative side, it is based on the Pollution Paradise Hypothesis^93^, which holds that some developed countries transfer high energy consumption and high pollution industries to some developing countries with relatively low environmental regulations through foreign direct investment, thereby reducing their pollution emissions^94^. According to the above hypotheses, the direction of foreign direct investment’s impact on the environment is primarily determined by the source and inflow channel of foreign direct investment. If the source of foreign direct investment is primarily knowledge-intensive industries based on advanced technology, the foreign direct investment will benefit the host country’s environmental conditions, and vice versa.

Regarding the influence mechanism between carbon emissions and foreign investment in typical Chinese cities, the results show that foreign direct investment has a higher degree of influence on the low-carbon level of cities, in addition to the indicators related to industrial pollutant emissions. Although Chinese typical cities are more likely to attract foreign investment because of their high-quality infrastructure conditions, stable and good market environment, and sufficient production materials. However, since China was still a developing country in this period, it reduced its investment in urban environmental regulation and relaxed the environmental regulation of overseas investment in the early stage of opening up to the outside world, which led to a certain degree of increase in total urban carbon emissions and environmental pollution. Therefore, FDI is still an important driver for the low-carbon level of typical cities in the eastern region. In addition, from the results, GDP is still the core driver of low-carbon levels in cities during this period, indicating that the dependence of cities’ economic growth on carbon-intensive industries is still high and the decoupling between their cities’ carbon emission level and economic growth has not been achieved.

## Conclusion

This study constructs a set of comprehensive evaluation index systems of low-carbon city multi-system from three dimensions of urban carbon emission, economy, and environment, measures and analyzes the trend of the low-carbon level of cities, and studies the influence mechanism of the multi-coupled system, aiming to find the current deficiencies in the construction of low-carbon cities in China and make suggestions for the next construction of low-carbon cities in China. From the analysis results, the percentage of cities with the low-carbon level in an upward or downward trend is relatively small, and the current trend of low-carbon levels in nearly two-thirds of the cities is still not significant. At the same time, in terms of the coupling and coordination between low-carbon levels and economic development, the temporal trend shows an increasing trend year by year, and the relationship between the two is in line with the “inverted U-shaped” characteristics. Spatially, it shows that the core cities of large urban agglomerations are at a high level, while the surrounding cities are at a relatively low level, forming a siphon effect of large cities, which has a negative externality to the coordinated development of typical cities around urban agglomerations to a certain extent. In addition, based on the analysis of the core drivers of low-carbon cities, we should continue to support the development of enterprises with high technological innovation capabilities, adjust the allocation of resources, give full play to the market regulation mechanism, and improve the regulatory system for foreign direct investment to guide more capital flows to low-carbon sustainable industries, to better accomplish the goal of low-carbon city construction and promote high-quality economic development.

## Data Availability

The datasets used and analysed during the current study available from the corresponding author on reasonable request.
